# Gold-Based Medicine: A Paradigm Shift in Anti-Cancer Therapy?

**DOI:** 10.3390/molecules23061410

**Published:** 2018-06-11

**Authors:** Chien Ing Yeo, Kah Kooi Ooi, Edward R. T. Tiekink

**Affiliations:** Research Centre for Crystalline Materials, School of Science and Technology, Sunway University. No. 5, Jalan Universiti, Bandar Sunway 47500, Malaysia; allyy@sunway.edu.my (C.I.Y.); samuelo@sunway.edu.my (K.K.O.)

**Keywords:** gold compounds, biological activities, cancer, metabolism

## Abstract

A new era of metal-based drugs started in the 1960s, heralded by the discovery of potent platinum-based complexes, commencing with cisplatin [(H_3_N)_2_PtCl_2_], which are effective anti-cancer chemotherapeutic drugs. While clinical applications of gold-based drugs largely relate to the treatment of rheumatoid arthritis, attention has turned to the investigation of the efficacy of gold(I) and gold(III) compounds for anti-cancer applications. This review article provides an account of the latest research conducted during the last decade or so on the development of gold compounds and their potential activities against several cancers as well as a summary of possible mechanisms of action/biological targets. The promising activities and increasing knowledge of gold-based drug metabolism ensures that continued efforts will be made to develop gold-based anti-cancer agents.

## 1. Introduction

Cancer, a disease that is derived from the mutation of genes, involves a series of alterations in cellular function and is often associated with persistent/uncontrolled inflammation (i.e., non-resolving inflammation [1]) in the tumour microenvironment that leads to the propagation of cancer [2]. Gold compounds have been used since ancient times, particularly in traditional Chinese, Egyptian and Indian medicines, where they were found to be effective in treating inflammation, infection and tuberculosis [3,4,5]. The discovery of the anti-cancer potential of cisplatin [cis-diamminedichloridoplatinum(II)] in the mid-1960s provided great impetus for the development of other metal-based compounds as pharmaceuticals [6]. While this hugely successful and widely used platinum-based complex is effective at inhibiting cancer cell activities—in particular head and neck and testicular cancers—through the formation of DNA-platinum adducts, this non-selective, DNA-targeted mechanism gives rise to several side effects, such as cardiotoxicity, nephrotoxicity and neurotoxicity [7,8,9,10]. Several biological studies have reported that gold compounds form comparatively weaker interactions with cellular DNA, indicating that gold(I) compounds, at least, carry out their bioactivities through a “DNA-independent mechanism” [11,12]. This review serves as an update of the developments associated with gold-based drugs/compounds achieved over the past decade, describing the current findings about gold compounds and their possible modes of action towards biological targets.

**Figure 1:**
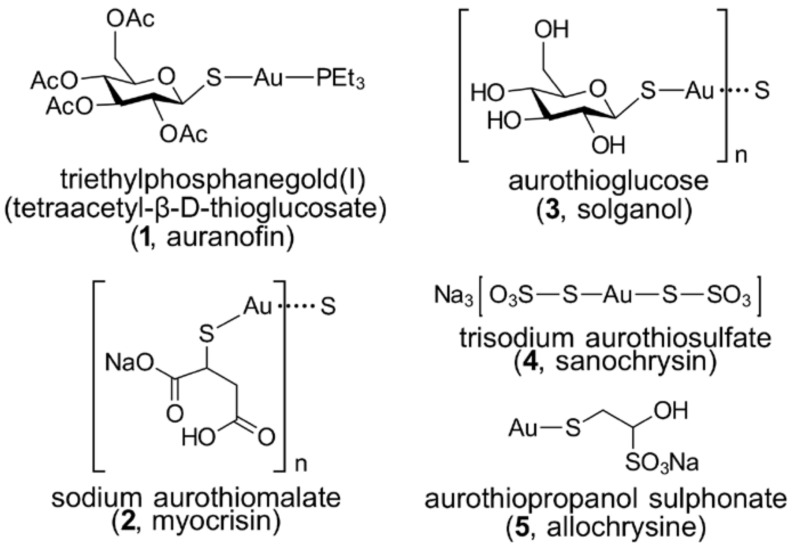
Chemical structures of gold drugs (**1**)–(**5**).

Following the longstanding use of gold drugs, in particular, for the treatment of rheumatoid arthritis (see reviews [3,13]), research continues to uncover the potential of gold compounds as anti-cancer agents (see reviews [11,14,15,16,17,18,19,20,21,22,23]). In many cases, new gold compounds have been prepared as derivatives based upon the “backbone” of clinically-available, gold-based drugs, as illustrated in Figure 1. The presence of phosphane and thiol ligands in auranofin, for example, stabilises the gold(I) centre, and new species have been designed to improve the efficacy of such compounds [14,15,16]. More recent efforts have focused upon the development of gold(III) complexes [17,24], through stabilisation of the higher oxidation states achieved with other, less labile ligands, bearing for example, carbon-, nitrogen- and oxygen-ligating atoms, heralds a bright future for gold-based drugs [14,16,25,26]. The stability of gold compounds in the physiological environment is essential as it accounts for the successful delivery of the active moiety to the targeted sites. In a recent review, the toxicological profiles and biological targets of prominent gold compounds were discussed. Gold compounds possess conspicuous therapeutic effects, and this warrants further investigation into their mechanisms of action to overcome adverse toxicity [27].

New impetus for the development of gold compounds as anti-cancer agents has come from (i) the observed anti-tumour activity of auranofin (**1**) and aurothiomalate (**2**) (Figure 1) and (ii) the development of stable gold(III) complexes that can mimic the mode of action of cisplatin. Auranofin and aurothiomalate are classic examples of “drug repositioning” [28,29], whereby a drug used for the treatment of a specific disease, in this case rheumatoid arthritis, is investigated for other therapies, in this case, cancer. Indeed, both auranofin (advanced or recurrent non-small cell lung cancer or small cell lung cancer; https://clinicaltrials.gov/ct2/show/NCT01737502; accessed, 4 April 2018) and sodium aurothiomalate (advanced non-small cell lung cancer; https://clinicaltrials.gov/ct2/show/NCT00575393; accessed on 4 April 2018) are undergoing clinical trials. The available gold(III) complexes were, for a long time, found to be unstable in the reducing environments of biological systems, making their use as putative drugs mimicking cisplatin problematic. However, with careful ligand design, gold(III) centres can be rendered relatively stable to reduction and hence, a resurgence has occurred in the investigation of gold(III) complexes as anti-cancer agents.

Herein, different classes of gold(I)/(III) compounds showing potential anti-cancer activity are surveyed along with an overview of what is known about the biological mechanisms of action promoted by gold compounds. The interested reader is referred to the original articles for details of chemical synthesis.

## 2. General Perspectives

In addition to the significant biological activities of gold compounds, the chemistry of gold has also been extensively studied owing to its distinctive chemistry [30,31,32]. In its elemental form, gold(0) appears as a bright-yellow metal with high resistance to harsh conditions, but is susceptible to reactions with aqua regia, during which oxidation to gold(III) occurs. In general, gold exists in several oxidation states, ranging from −I to +V, with gold(I) and gold(III) being the most common forms employed for biological studies. Relativistic effects, leading to the stabilisation of the 6s valence electrons and the destabilisation of the 5d electrons, give rise to many of the physical and chemical properties of gold, including its distinctive colour [32,33].

Gold(I), with a d^10^ closed-shell configuration, generally gives rise to two-coordinate, linear compounds, which are by far the most commonly observed coordination geometry, as well as three-coordinate, trigonal and four-coordinate, tetrahedral coordination geometries [34]. Being a “soft” metal centre, gold(I) has a pronounced tendency to form stable complexes with easily polarisable soft donor atoms, such as sulphur and phosphorus [34]. Gold(III), on the other hand, usually forms a tetra-coordinate, square planar geometry with a preference for hard donor atoms, including oxygen and nitrogen, due to the “hard” nature of gold(III) compared with gold(I) [34]. The presence of the same electronic configuration and structural characteristics as cisplatin prompted the investigation of gold(III) complexes as anti-tumour agents but was initially restrained because such complexes are susceptible to reduction in the biological environment [25]. However, many significant advances with gold(III) complexes have been made in recent years with careful selection of coordination geometries (see below).

In the modern era of pharmacy, the first gold compound reported to display biological properties was a potassium gold(I) cyanide salt, K[Au(CN)_2_] (6), prepared by Robert Koch for the investigation of anti-tubercular activities [3]. This important discovery formed the basis for investigating the pharmacological activities of gold compounds. “Chrysotherapy” is the term that refers to the use of gold formulations in medicine, in particular for the treatment of joint pain and inflammatory diseases, such as rheumatoid arthritis [14]. The early medicinal benefits of gold compounds were limited to inflammatory diseases and hence, they were initially classified as Disease Modifying Anti-Rheumatic Drugs (DMARDs) [15,35]. As time progressed, it was noticed that the recipients of chrysotherapy for arthritis exhibited lower evidence of malignancy rates and thus, it was hypothesised that gold compounds might possess anti-cancer effects [34,36,37,38,39]. In general, gold compounds tend to target cancer cells, and the onset of cell death mechanisms occurs as early as two hours after receiving treatment [40].

To date, a wide range of gold compounds with promising anti-cancer properties have been designed by taking into account the following: (i) having chemical structures/properties resembling gold(I) anti-arthritic agents; (ii) gold(III) complexes analogous to platinum(II) complexes, sharing a common d^8^ closed-shell configuration; and (iii) incorporating molecules with known anti-cancer properties to prepare gold(I) and gold(III) complexes with enhanced activities—the “Trojan-horse” strategy [34]. The prominent classes of gold compounds currently being investigated for anti-tumour potential are outlined below in Section 3.

## 3. Gold(I) and Gold(III) Compounds with Promising Anti-Cancer Potential

There are several classes of gold compounds that have been investigated for anti-cancer activity and the following sections provide a brief overview of their chemistry and activity in order to highlight the different types of compound that have been screened: phosphanegold(I) compounds (3.1), gold thiolates (3.2), gold porphyrins (3.3), gold compounds with bipyridyl-type ligands (3.4), and organogold species—gold N-heterocyclic carbenes (3.5), cyclometallated gold complexes (3.6) and gold alkenyls (3.7).

### 3.1. Phosphanegold(I) Compounds

The presence of a phosphane ligand in gold thiolates (See Section 3.2) generally results in efficacious activity but, at the same time, is highly dependent on the judicious selection of phosphane ligands. This is also true for non-thiolate species. Thus, the increased lipophilicity in chloridophosphanegold(I) compounds prepared from simple phosphanes, e.g., PMe_3_, PEt_3_, P(t-Bu)_3_ and PPh_3_ [7], as well as water-soluble phosphanes, namely P(m-C_6_H_4_SO_3_Na)_3_, P(R)(m-C_6_H_4_SO_3_Na)_2_ and P(R)_2_(m-C_6_H_4_SO_3_Na) with R = n-Bu and cyclopentyl [41], leads to enhanced biological properties. However, the high lipophilicity of bis-chelated, tetrahedral [Au(dppe)_2_]^+^ (**7**) [42] was shon to induce severe hepatotoxicity owing to mitochondrial dysfunction, which later prompted the preparation of [Au(d2pypp)_2_]Cl (**8**) with reduced lipophilicity by Filipovska, Berners-Price and colleagues [43] (dppe is Ph_2_PCH_2_CH_2_PPh_2_ and d2pypp is 1,3-bis(di-2-pyridylphosphino)propane, (2-pyr)_2_P(CH_2_)_3_P(2-pyr)_2_). The above indicates the importance of fine-tuning the lipophilic and hydrophilic properties of the compound in order to influence its cellular uptake and tumour selectivity. For example, [Au(d2pypp)_2_]Cl (**8**) is less lipophilic than [Au(dppe)_2_]Cl (**7**) and is selectively delivered to the mitochondria of breast cancer cells as evidenced from the subcellular distribution of gold identified in the cytoplasm [43]. There is evidence to suggest that the mechanism of action of gold compounds is, therefore, associated with the inhibition of thiol-containing protein families [43,44]. The screening of another series of [(diphosphane)(AuCl)_2_] species and bis-chelated [(diphosphane)_2_Au]X gold(I) compounds, prepared from imidazole- and thioazole-based, diphosphane-type ligands against human ovarian (A2780 sens and A2780 cisR), human leukaemia (K562), human colon carcinoma (Hct116) and a rat hepatoma cell line (H4IIE) also revealed a significant correlation between the compounds’ levels of lipophilicity and cytotoxicity [45] (diphosphane = bis(diphenylphosphane)-methane (dppm), -ethane (dppe), etc). It was reported that only compounds with intermediate lipophilicity showed a significant level of cytotoxicity against the tested cell lines. In a study of compounds bearing phosphane ligands with various levels of denticity, e.g., Ph_3_PAuCl (**9**), [Au_2_(dppm)]Cl_2_ (**10**) and [Au_3_(μ-dpmp)]Cl_3_ (**11**), against eight human cancer cell lines, the results implied that there was no direct correlation between the cytotoxicity and the phosphane ligand’s denticity [46] (dpmp is Ph_2_PCH_2_P(Ph)CH_2_PPh_2_). Rather, both mono- and bi-nuclear phosphanegold(I) compounds exhibited comparable activities, although the mononuclear derivative showed better cytotoxicity in certain cell lines. The trinuclear species on the other hand exerted the lowest effect among the three species trialled against the screened cancer cell lines [46].

### 3.2. Gold Thiolates

**Figure 2:**
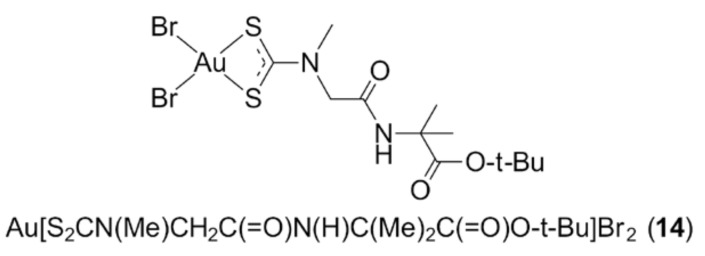
Chemical structure of (**14**).

Notwithstanding the generally poorer stability of gold(III) complexes under physiological conditions owing to their high hydrolysis rates and significant reduction potential, the interest in gold(III) complexes stems from the substantial anti-cancer properties of cisplatin (and second-generation derivatives) which shares similar chemical properties and preference for square-planar coordination geometries, due to its common d^8^ electronic configuration. Sulphur-containing ligands are often introduced to improve the stability of gold(III) complexes, with dithiocarbamates (^−^S_2_CNRR’) receiving considerable attention due to the substantial stabilising effect of these bidentate chelating molecules, owing to the significant contribution (40%) of the dithiolate canonical form (i.e., ^2−^S_2_C=N^+^RR’) to the electronic structure of the ligand. The gold(III) dithiocarbamate complexes, Au(S_2_CNMe_2_)Cl_2_ (**12**) and Au[S_2_CN(Me)CH_2_C(=O)OEt]Br_2_ (**13**) [47,48], have shown outstanding anti-tumour activities both in vitro and in vivo. The former induced an overall 85% reduction of implanted human prostate tumours (xenografts) in nude mice accompanied by minimal systemic toxicity, while (**13**) induced better inhibitory properties and reduced acute toxicity in three transplantable murine tumour models, namely solid Ehrlich carcinoma, Ehrlich ascites and Lewis lung carcinoma, compared to cisplatin. Fregona and colleagues took an additional step and tailored gold(III) dithiocarbamate derivatives further by introducing dipeptide substituents, with the aim of achieving better intracellular drug transfer and tumour specificity by targeting peptide transporters [49,50]. Among the complexes screened, the dithiocarbamate ligand functionalised with the dipeptide derived from the condensation of sarcosine and 2-aminoisobutyric acid, Au[S_2_CN(Me)CH_2_C(=O)N(H)C(Me)_2_C(=O)O-t-Bu]Br_2_ (**14**) (Figure 2), presented exceptional in vitro cytotoxic effects, surpassing cisplatin by inducing apoptosis to prostate (PC3 and DU145) and ovarian adenocarcinoma cisplatin-resistant (C13) cancer cell lines. On the other hand, this complex caused late apoptosis/necrosis to parent ovarian adenocarcinoma cisplatin-sensitive 2008 cells and Hodgkin’s lymphoma L540 cells. The mechanism of action of (**14**) on the triple-negative human breast cancer (TNBC) cell line, MDA-MB-231, was later elucidated, in vitro and in vivo, and was revealed to mediate apoptotic cell death, with proteasome being the major target [50].

Recently, it was suggested that [Au(S_2_CNRR’)_2_]^+^ species are responsible for interaction with sulfanyl-containing substances in biological environments as well as for the resulting cytotoxicity of these complexes [51]. However, it is worth highlighting that a few other factors, such as the hydrophobicity of the dithiocarbamate ligand and the accessibility of the gold(III) complexes to the biological targets also have considerable impacts upon inhibition activities [49,51,52]. A series of phosphanegold(I) dithiocarbamates provided evidence that compounds with greater steric hindrance exhibit reduced cytotoxicity effects against human lung (A549), cervical (HeLa) and colon (HCT15) cancer cell lines [53]. Gold(I) thiolate derivatives are also being investigated for anti-cancer potential.

Despite the fact that the linear P–Au–S moiety confers stability to the gold(I) centre, the selection of phosphane ligand has also proven to be equally important to allow better selectivity and anti-cancer properties. In a series of compounds with the general formula, R_3_PAu[S_2_CN(iPr)CH_2_CH_2_OH], for R = Ph (**15**), Cy (**16**) and Et (**17**), each displayed a different mechanism of cell death with the R = Ph derivative (**15**) being the most potent and inducing apoptosis in MCF-7R breast cancer cells, while necrosis was observed for R = Cy (**16**) and Et (**17**) derivatives [54]. While both monophosphane and long chain bisphosphane ligands gave rise to stable phosphanegold(I) dithiocarbamates, Darkwa and co-workers reported the outstanding activities of various Au_2_(S_2_CNRR’)_2_(Ph_2_P(CH)_6_PPh_2_) compounds against a broad panel of cancer cell lines, with tumour specificity outdoing other counterparts with monophosphanes and diphosphanes with shorter alkyl bridges [55]. Various other phosphanegold(I) thiolates featuring the linear P–Au–S arrangement also displayed promising anti-cancer properties, including those derived from *O*-alkylthiocarbamides [56] and five- and six-membered heterocycles [57,58], as well as all-thiolate salts of cyclic thioureas [59]. As a general observation, gold thiolate compounds presented better activities than cisplatin and the presence of thiolate ligands proved to be essential for improved efficacy [51].

### 3.3. Gold Porphyrins

**Figure 3:**
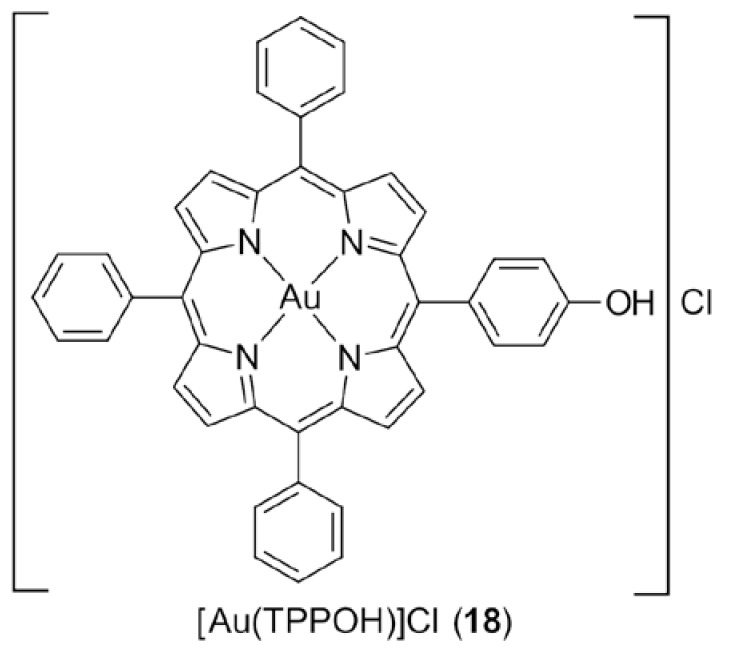
Chemical structure of (**18**).

The robust chelating ability of porphyrin ligands provides a rigid scaffold for its metal complexes and thus, it is not surprising that porphyrin is an excellent ligand for the formation of stable gold(III) complexes. Recently, Che and colleagues highlighted that the coupling of gold(III) with porphyrin ligands plays an essential role in the inhibition of HeLa cervical cancer cells (Hsp60) while other non-porphyrin gold(III) and platinum(II) porphyrin have complexes exhibited relatively poorer activities [60]. An important member of the series of gold(III) porphyrin complexes that were investigated, [Au(TPPOH)]Cl (**18**) (Figure 3), has been subjected to extensive, ongoing studies and has demonstrated remarkable stability under physiological (reducing) conditions. In terms of cytotoxicity, (**18**) exhibited better potency cf. cisplatin in vitro against a large panel of human cancer cell lines, including multi-drug resistant human oral epidermoid carcinoma (HSC-3), cisplatin-resistant nasopharyngeal carcinoma (HNE-1), hepatocellular carcinoma (HepG2), neuroblastoma (SH-SY5Y), colon carcinoma (HT-29), nasopharyngeal carcinoma (HK-1), and cisplatin-sensitive (A2780) and cisplatin-resistant (A2780cis) ovarian cancers cells, and, in vivo, it inhibited the tumour growth of mice bearing nasopharyngeal carcinoma (NPC) [61,62,63,64,65,66,67,68]. Further, work was directed to address the possible mechanisms behind therapeutic resistance encountered in neuroblastoma cells. The activation of protein kinase B (PKB), also known as Akt, by (**18**) before the onset of apoptosis was postulated as the key reason behind the induction of the encountered resistance [69]. This hypothesis was strengthened by the fact that the anti-cancer activity of (**18**) in neuroblastomas was enhanced after the introduction of Akt/protein kinase B signalling inhibitor (API-2). The study on gold(III) porphyrin complexes as putative anti-cancer agents was further extended to delineate the structure-activity relationship of these complexes against nasopharyngeal (SUNE1) cells [70]. Generally, gold(III) porphyrin derivatives with higher levels of lipophilicity were shown to give rise to enhanced cytotoxicity. It is also noteworthy that the anti-cancer efficacy of gold(III) porphyrin complexes can be heightened through their encapsulation within mesoporous silica nanoparticles [71].

### 3.4. Gold Compounds with Bipyridyl-Type Ligands

**Figure 4:**
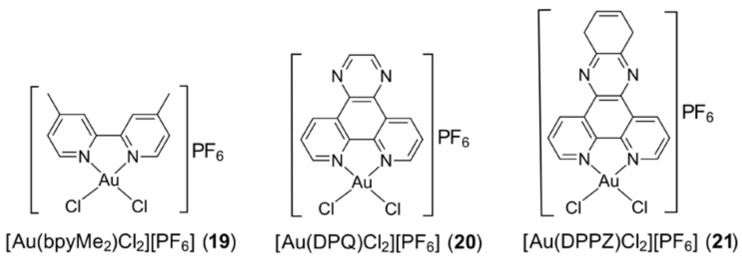
Chemical structures for (**19**)–(**21**). Abbreviations: bpyMe_2_ is 5,5′-dimethyl-bipyridine, DPQ is 1,4,8,9-tetraazatriphenylene and DPPZ is dipyrido[3,2-a:2′,3′-c] phenazine.

Gold(III) complexes bearing bipyridyl-type molecules have displayed promising anti-cancer properties as presented, for example, in the work by Casini et al. [72] and Palanichamy et al. [73]. Three key exemplars based on substituted 2,2′-bipyridine (bpy), namely [Au(bpyMe_2_)Cl_2_][PF_6_] (**19**), [Au(DPQ)Cl_2_][PF_6_] (**20**) and [Au(DPPZ)Cl_2_][PF_6_] (**21**), are depicted in Figure 4. The cations were evaluated against, for example, the human ovarian carcinoma cell line (A2780) and the cisplatin-resistant variant (A2780cisR) (**19**); and human ovarian adenocarcinoma cells (A2780) and cisplatin-resistant ovarian cancer cells (A2780CP70) (**20**) and (**21**). The gold(III) complexes were proven to react with amino acids rather than nucleic acids, in contrast to cisplatin which is known to target nucleic acids [72]. Further, the gold(III) complexes induced apoptosis with lower cytotoxic effects exerted on normal non-cancerous cells, compared to that induced by cisplatin [73].

The choice of the bipyridyl-type ligand has an influence on a given complex’s cytotoxicity, and this is also evident for the 1,10-phenanthroline derivative [(^sec-butyl^phen)AuCl_3_] (**22**) [74], a five-coordinate species with a phen-N in an apical position; the sec-butyl substituents occupy positions adjacent to the phen-nitrogen atoms. Complex (**22**) showed enhanced cytotoxicity against five human tumour cell lines, viz. non-small cell lung cancer squamous cell carcinoma (H1703) and adenocarcinoma (A549), primary squamous cell carcinoma (Tu212), primary squamous cell carcinoma (Tu686) and lymph node metastasis of squamous cell carcinoma (886LN) which correlated with its greater stability in the presence of the biological reductant, glutathione, as compared to the methyl/bipyridyl analogue, [(^methyl^byp)AuCl_2_]Cl (**23**). Unfortunately, it transpired that the in vitro results for (**22**) did not translate to good in vivo activity against a xenograft tumour in a murine model (head and neck squamous cancer cell lines, Tu212), possibly due to significant interactions with serum albumin [74].

**Figure 5:**
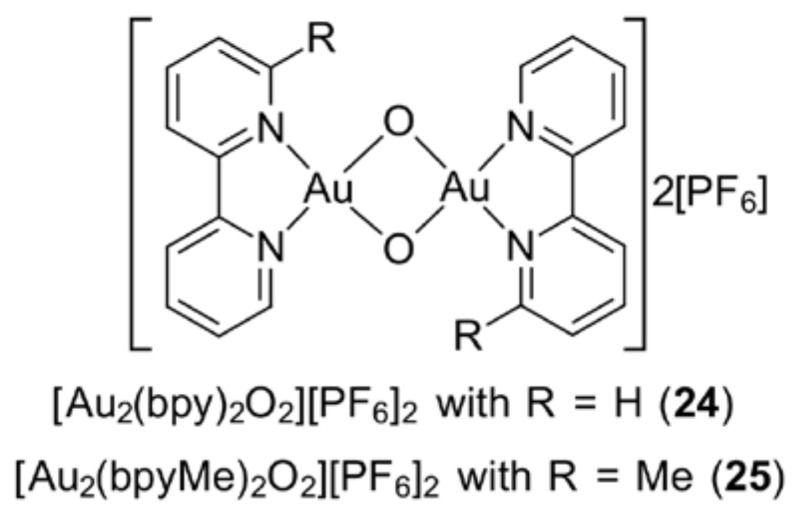
Chemical structure for (**24**) and (**25**).

An alternative method to improve the cytotoxicity profile of gold(III) pyridyl derivatives is through the replacement of chloride ligands with bridging oxygen atoms within the bipyridyl-like ligand molecules, as exemplified for [Au_2_(bpy)_2_O_2_][PF_6_]_2_ (**24**) in Figure 5. Their anti-proliferative effects were evaluated against three human tumour cell lines—breast cancer (MCF-7), cervical cancer (HeLa) and hepatic carcinoma (HepG2)—and three non-tumorigenic cell lines—human renal cortical epithelial (HRCE), human keratinocyte (HaCaT) and rat cardiomyoblast (H9c2). A reduced toxicity effect was exerted on the non-tumorigenic cell lines [75,76]. Referring to Figure 5, the derivative with R = Me (**25**) was encapsulated in an apoprotein-based ferritin that did not contain iron, with a protein:gold ratio of 75:1, and the resultant species was tested on the same cells as above. The MTT assay revealed that the encapsulated species exhibited reduced cytotoxicity towards cancerous cells (e.g., a two-fold reduction in potency against HepG2) but had significantly reduced toxicity towards normal HRCE, HaCaT and H9c2 cells as compared to the original molecule, by nearly 30-, five- and 15-fold, respectively [76].

In a series of comparative studies, the relative efficacy of binary complexes with phosphane derivatives pointed to the importance of having a phosphane ligand in the compound. For example, bis(saccharinate)gold(I) cations are less potent against human ovarian carcinoma (A2780S) cell line sensitive to cisplatin and its cisplatin-resistant counterpart (A2780R) than ternary phosphanegold(I) saccharinate analogues [39]. Studies of phosphanegold(I) pyrazolates and imidazolates against human breast (MCF-7), lung (A549), cervical (A431), colon (LoVo and LoVo MDR) and ovarian (2008 and C13) cancer cell lines [77], and phosphanegold(I) 1,3-benzodiazolates functionalized with 2-pyridyl groups against human ovarian carcinoma cells resistant to (A2780/R) or sensitive to cisplatin (A2780/S) [78], have proven the advantage of having triphenylphosphane as the phosphane ligand over water soluble 1,3,5-triaza-7-phosphaadamantane.

### 3.5. Gold N-Heterocyclic Carbenes

**Figure 6:**
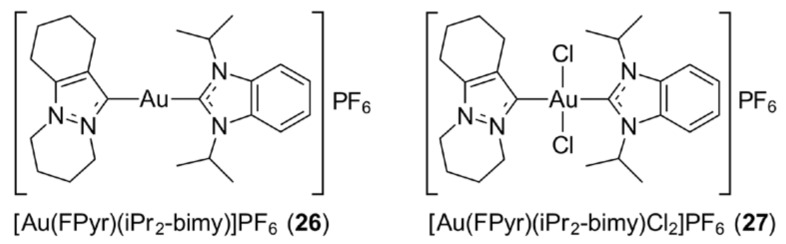
Chemical structures of (**26**) and (**27**). Abbreviations: FPyr is 1,3-diisopropylbenzimidazolin-2-ylidene and iPr_2_-bimy is 1,2,3,4,6,7,8,9-octahydropyridazino[1,2-a]indazolin-11-ylidene.

*N*-heterocyclic carbene (NHC), being a strong σ-donor ligand, renders gold(I) compounds with enhanced levels of stability under physiological conditions. The early emphasis on this class of compound was dominated by gold(I) species but later, gold(III) NHC complexes attracted considerable interest. The gold(III) congeners can be prepared by treating a gold(I) NHC precursor with PhICl_2_, affording a high yield of gold(III) species [79]. Alternatively, Me_2_SAuCl can be used a source of gold(III) as this readily undergoes disproportionation, whereby some of the original gold(I) is oxidised to gold(III), while at the same time, some is reduced to gold(0) [80,81,82]—the latter can be reused after isolation. A broad range of gold(I) monocarbene, bis(carbene), hetero-bis(carbene) and their respective gold(III) analogues were prepared by Huynh and co-workers [79]; some representative chemical structures are depicted in Figure 6—the hetero-bis(carbene) compounds of gold(I) (i.e., [Au(FPyr)(iPr_2_-bimy)]PF_6_ (**26**)) and gold(III) (i.e., [Au(FPyr)(iPr_2_-bimy)Cl_2_]PF_6_ (**27**)). The gold(I) and gold(III) compounds were targeted to delineate their in vitro levels of cytotoxicity towards the non-small cell lung cancer (NCI-H1666) cell line. Overall, cationic gold(I) and gold(III) bis(carbene) species demonstrated better cytotoxicity effects cf. the monocarbene analogues and cisplatin, with hetero-bis(carbene) compounds having superior activity. Gold(I) carbenes were generally more active than their gold(III) congeners, and it is suggested the gold(III) carbenes were, in fact, reduced to gold(I) species [80,81,82]. In thioredoxin reductase (TrxR) inhibition studies on a series of gold(I) and gold(III) NHC compounds, also bearing halides and thiolate ligands, the significant TrxR inhibition was believed to contribute largely to their prominent anti-proliferative effects against a colon carcinoma (HT-29) cell line [83]. Gold(I) species present greater TrxR inhibitory effects compared to their gold(III) congeners, indicating that intact Au–NHC is likely responsible for the biological properties [83,84]. Attempts to interact potent gold(I)–NHCs with cytochrome c and lysozyme—chosen as model proteins—did not result in protein–gold adducts despite long incubation periods [85]. The selectivity of gold–NHCs complexes against cancer cells, and, indeed, other metal–carbene complexes, is most likely related to their delocalised lipophilic cationic (DLC) behaviour which favours their accumulation in cancer cells due to their relatively high mitochondrial membrane potential [86,87,88,89]. A very recent review described the medicinal applications of gold(I)/(III) complexes bearing N-heterocyclic carbenes [90].

### 3.6. Cyclometallated Gold Complexes

**Figure 7:**
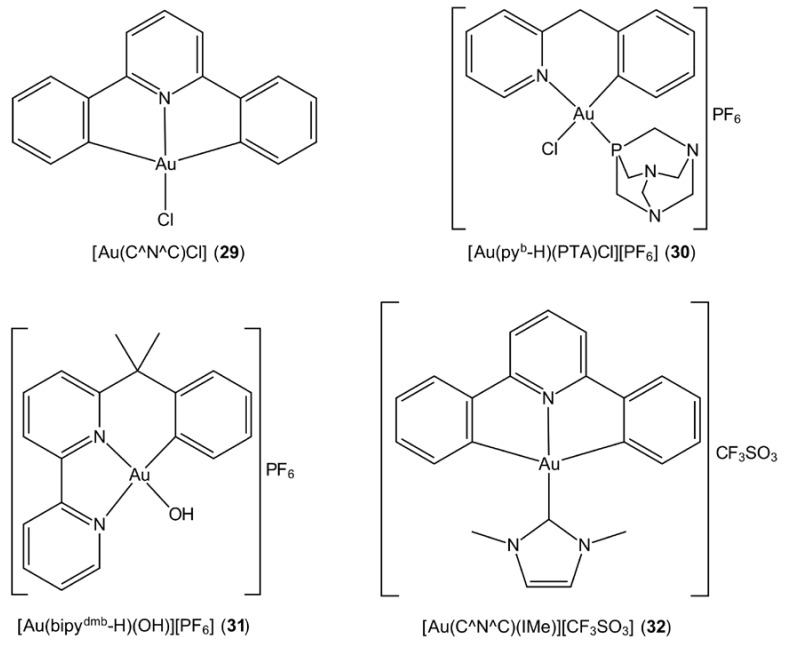
Chemical structures of (**29**)–(**32**). Abbreviations: C^N^C is the bi-cyclometallated di-anion derived from 2,6-diphenylpyridine, py^b^-H is the cyclometallated ligand derived from 2-benzylpyridine, bpy^dmb^ is the cyclometallated anion derived from 6-(1,1-dimethylbenzyl)-2,2′-bipyridine and IMe is *N*,*N*’-dimethylimidazolium.

Cyclometallation contributes to the stability of gold(III) complexes through the coordination of a ring-carbon atom within a chelating ligand which results in the formation of a strong covalent Au‒C bond. Che and co-workers reported mono-, bi- and tri-nuclear cyclometallated gold(III) complexes with the general formula [Au_m_(C^N^C)_m_L]^n+^, where L is chloride or a neutral ligand, such as phosphane and pyridine [91] (see Figure 7 for the chemical structure of the prototype complex, [Au(C^N^C)Cl] (**28**)). These complexes possess promising stability with no evidence of demetallation upon treatment with glutathione. Further, it has been proposed that the [Au(C^N^C)]^+^ moieties enhance the stability of the gold–phosphane ligand, paving the way for the possibility for [Au(C^N^C)]^+^ to function as a carrier for biologically active molecules such as phosphane [91]. Owing to the prominent in vitro cytotoxicity originally reported for [(μ-dppp)Au_2_(C^N^C)_2_](CF_3_SO_3_)_2_ (**29**) [91], this complex was revisited seven years later together with other binuclear derivatives for the assessment of anti-tumour efficacy in a nude mice model bearing human hepatocellular carcinoma (PLC) cells [92] (dppp is 1,2-bis(diphenylphosphino)propane, Ph_2_P(CH_2_)_3_PPh_2_). In that study, it was discovered that the nature of the bridging phosphane ligand influenced the compound’s potency. The binuclear cyclometallated gold(III) complex bridged by a dppp showed the greatest anti-cancer properties among the series, while changing of the bridging linker resulted in reduced efficacy. This particular dimeric complex also demonstrated better cytotoxic activity compared to the monomeric derivative and other related compounds with the phosphane ligand replaced by carbene substituents. The crucial role of phosphane ligand in enhancing in vitro toxicity was further demonstrated by the presentation of the most prominent toxicity profile among the derivatives by [Au(py^b^-H)(PTA)Cl][PF_6_] (**30**) (Figure 7) [93]. Different studies have revealed that both cyclometallated gold(III) complexes with C^N- and C^N^N-type ligands, e.g., [Au(bpy^dmb^-H)(OH)][PF_6_] (**31**), (Figure 7) exhibit enhanced stability under physiological conditions compared to those coordinated by N-donor ligands and that this stability accounts for their reactivity towards nucleobases [94]. As reported by Che and colleagues, the incorporation of N-heterocyclic carbenes into cyclometallated C^N^C gold(III) complexes imparts stabilisation and improves solubility. In addition to the above, the screening of the in vivo anti-cancer properties of the [Au(C^N^C)(IMe)][CF_3_SO_3_] (**32**) complex (Figure 7) in a nude mice bearing hepatocellular carcinoma (PLC) resulted in significant tumour size reduction with no apparent toxic side effects [95].

### 3.7. Gold Alkynyls

**Figure 8:**
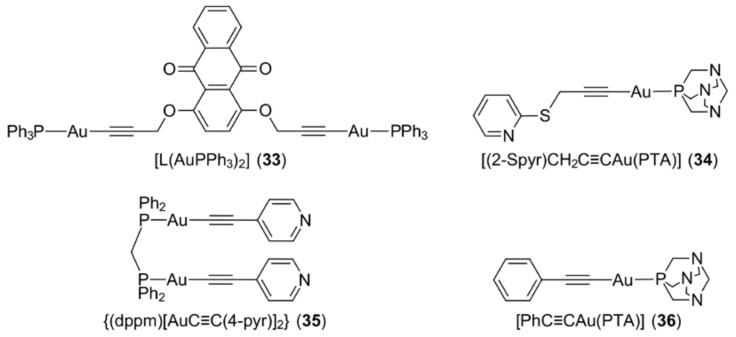
Chemical structures of (**33**)–(**36**). Abbreviation: L is the di-anion derived from 1,4-bis(ethynyloxy)anthracene-9,10-dione.

Alkynyl ligands with a triply bonded di-carbon (‒C≡C‒) fragment, coordinate gold(I) to form organogold(I) compounds featuring an Au–C bond. Knowledge of the biological properties of this type of organogold(I) compound remains relatively scarce but the revealed promising anti-cancer properties ensure continued investigation. Mono- and di-gold(I) triphenylphosphane complexes derived from 1,4-, and 1,8-dialkynyloxyanthraquinones have been found to possess cytotoxicity against four cancer cell lines, namely, breast adenocarcinoma (MCF7), lung adenocarcinoma (A549), prostate adenocarcinoma (PC3) and colon adenocarcinoma (LOVO) [96] (see Figure 8 for an exemplar of a binuclear complex, i.e., [L(AuPPh_3_)_2_] (**33**)). These compounds were shown to exhibit greater toxicity towards MCF7 cancer cells which have a greater mitochondrial mass and thus, it was proposed that mitochondria are the key intracellular target. In a different study, a series of gold(I) alkynyl compounds functionalised with thiopyridine-type substituents and with water soluble 1,3,5-triaza-7-phosphaadamantane (PTA), e.g., [(2-Spyr)CH_2_C≡CAu(PTA)] (**34**) (Figure 8), induced apoptosis in human colon cancer cell lines (Caco-2: PD7 and TC7 clones) as well as moderately inhibiting the tumour growth in athymic (i.e., lacking a thymus gland) nude mice, with no significant toxicity observed in the organs (kidney and liver) [97]. It is noteworthy that these complexes could possibly induce cytotoxicity by acting as pro-drugs, as time-dependent spectroscopy showed their progressive transformation (aquation) in physiological medium. Generally, high lipophilicity often correlates with poor solubility and therefore, hampers the biological properties of gold-based drugs. This generalisation holds true in the case of phosphane-bridged binuclear gold(I) alkynyl compounds [98], e.g., {(dppm)[AuC≡C(4-pyr)]_2_} (**35**) (Figure 8), but not so in gold(I) alkynyl compounds, such as [PhC≡CAu(PTA)] (**36**) [99,100], Figure 8. The latter results highlight the importance of the presence of reasonable lipophilicity to enable the transportation of gold(I) alkynyl compounds in blood.

## 4. Other Approaches

**Figure 9:**
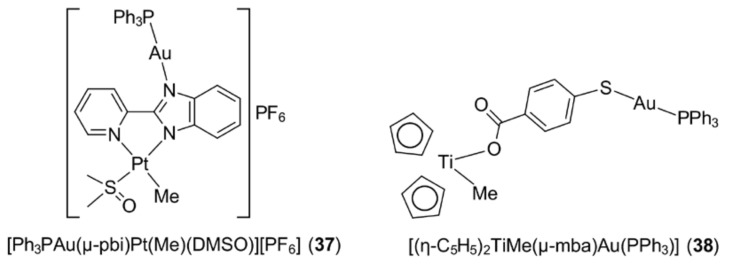
Chemical structures of (**37**) and (**38**). Abbreviations: pbi is the anion derived from 2-(pyridin-2-yl)-1H-1,3-benzodiazole and mba is the di-anion derived from 4-mercaptobenzoic acid.

The significant toxic side effects and cancer resistance associated with cisplatin and second generation platinum drugs coupled with the sometimes poor stability of gold compounds in the physiological environment, have prompted various strategies aimed at improving the efficacy of metal-based drugs. One of the alternatives includes designing compounds bearing two or more metal centres to exploit possible synergistic effects out of the combination. Recently, the incorporation of a [Ph_3_PAu]^+^ moiety into a platinum complex resulted in the formation of [Ph_3_PAu(μ-pbi)Pt(Me)(DMSO)][PF_6_] (**37**) (Figure 9) which showed improved anti-proliferative effects against the cisplatin-sensitive (A2780/S) and cisplatin-resistant (A2780/R) human ovarian cancer cell lines [101]. However, additional tests were performed, with the results suggesting that the heterobimetallic complex in fact simply manifested an additive effect rather than producing a synergistic effect. The anticipated synergistic effect for heterobimetallic complexes was observed in several gold(I)–ruthenium(II) and gold(I)–titanium(IV) complexes [102,103,104,105,106,107]. It is worth highlighting that these heterobimetallic complexes, in general, exhibited enhanced cytotoxic effects with improved stability and solubility, while being more selective towards cancer cells and less toxic to non-tumorigenic human cell lines. An in vivo study performed on [(η-C_5_H_5_)_2_TiMe(μ-mba)Au(PPh_3_)] (**38**) (Figure 9) in Caki-1 xenografted mice further supported the synergism of heterobimetallic strategy, i.e., impressive tumour shrinkage without significant side effects [107]. Gold nanoparticles (GNPs) on the other hand offer a safe and promising delivery scaffold for anti-cancer drugs, but as the focus of this review is molecular gold compounds, the details of GNPs will not be discussed here [108,109,110]. Suffice to mention that, in general, several factors account for the effectiveness of GNPs for drug delivery, including pH and shape [111,112], and GNPs not only function as drug carriers, they can also play a crucial role in mediating anti-cancer activity [113,114].

## 5. Gold Compounds and Their Biological Mechanisms of Action

In some cases, gold compounds inhibit the proliferation of cancer cells by targeting mitochondrial activities which induces the disruption of the electron transport chain and the respiration cycle accompanied by changes in mitochondrial membrane permeability [115]. The other general modes of action exhibited by gold compounds include direct DNA damage, cell cycle arrest, topoisomerase inhibition, specific kinase inhibition and inhibition of other transcriptional factors, to name a few [116,117,118,119]. These molecular targets have attracted interest as their mutation, over-activation and/or dysfunction are identified as the key factors leading to carcinogenesis. Upon DNA damage or protein/enzyme inhibition by gold complexes, apoptosis is triggered, thereby leading to the elimination of the cancer cell population [120]. In this section, several consequences of gold-based therapy are discussed: direct DNA damage (5.1), inhibition of topoisomerase (5.2), mitochondria and the inhibition of their function (5.3), the mitochondrial-dependent apoptosis pathway (5.4) and inhibition of thioredoxin (Trx) (5.5).

### 5.1. Direct DNA Damage

Cisplatin and its second generation counterparts, belong to one of the leading classes of metal complexes employed in chemotherapy for cancer treatment, are thought to exert their pharmacological activities via the formation of adducts with cellular nuclei acids—either DNA or RNA—an observation that prompted many studies on the mechanisms of anti-cancer activity exerted by other metal-containing species [121]. Being at the centre of genetics, DNA regulates most biochemical processes. Cancer cells generally carry extra copies of DNA as a consequence of abnormal DNA replication, which gives rise to the feasibility of targeting DNA by therapeutics [122,123]. The formation of DNA adducts alters DNA structure and subsequently, renders cancer cells incapable of proceeding to the different checkpoints of the cell cycle for replication, resulting in targeted cancer cell death [124]. Although cancer cell death can be achieved effectively, chemotherapy is often non-selective and targets the DNA of normal cells as well to causing undesirable side effects [125,126]. Many efforts are, therefore, directed towards identifying metal complexes with greater selectivity, such as gold(I) and, especially, gold(III) complexes with a d^8^ electronic confirmation as found in platinum(II) anti-cancer drugs, although generally, they are reported to have a poorer DNA binding ability [11,127,128,129]. Further, gold(III) complexes, bearing higher amounts of positive charge than their gold(I) congeners, should possess a greater affinity towards the relatively negatively charged DNA [130,131]. In general, the “hard” nature and square-planar geometry of gold(III) complexes should facilitate the covalent bonding of the gold centre to nucleobase nitrogen atoms or phosphate groups, forming a DNA–Au adduct to be followed by denaturation and fragmentation of DNA [131,132,133]. In the event of formation of lesions on the DNA, a cell death mechanism is then initiated to remove these DNA-injured cells, preventing these DNA-damaged cells from further replication.

**Figure 10:**
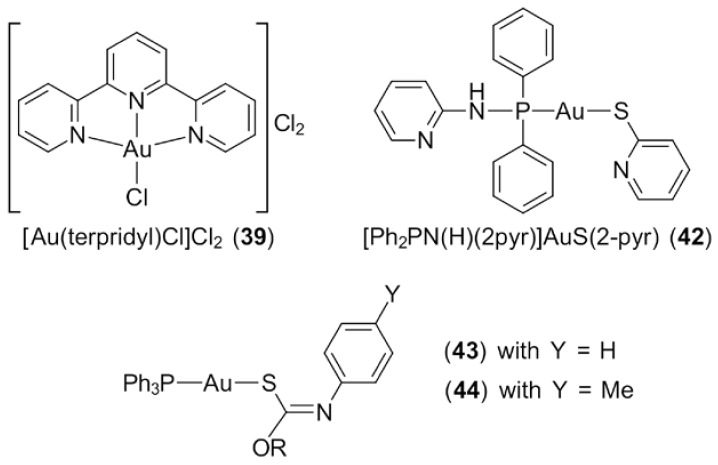
Chemical structures of (**39**), (**42**)–(**44**).

While platinum-based complexes possess a greater tendency to permanently intercalate into DNA and produce irreversible metal–DNA adducts, gold(III) complexes, on the other hand, present reversible activities towards DNA and are highly influenced by the nature of gold-bound ligands [12,134,135]. As reported in DNA-binding studies of gold(III) complexes using calf-thymus DNA as the experimental model, complexes bearing pyridyl, bipyridyl and polyamine ligands form weak and reversible interactions towards DNA as DNA undergoes self-repair processes [134,136]. However, the coordination of terpyridine ligands to gold(III), such as archetypal [Au(terpridyl)Cl]Cl_2_ (**39**) (Figure 10) (cf. the neutral cyclometallated species shown in Figure 7) was shown to result in stronger and irreversible intercalation comparable to DNA platination [130,137]. A series of gold(III) dithiocarbamates (i.e., Au(S_2_CNMe_2_)X_2_ (**40**) and Au[S_2_CN(Me)CH_2_C(=O)OEt]X_2_ (**41**) for X = Cl or Br) was shown to form gold(III)–DNA adducts with faster kinetics than those exhibited by cisplatin, leading to cytotoxic effects and eventually, cell death mechanisms [132]. In addition to the above, the coordination of dithiocarbamate ligands to gold(III) was shown to enhance the stabilisation of gold(III) by preventing their reduction in the mammalian environment (See Section 3.2), thus increasing their anti-cancer activity towards cisplatin-resistant cancers, such as ovarian cancer and prostate cancer [47,138]. Ronconi et al. further suggested that dithiocarbamates improve the selectivity of the complexes towards cancer cells by inhibiting interactions between the gold centre and the thiol groups of renal enzymes, thus eliminating cisplatin-like induced nephrotoxicity [24].

Gold(I) compounds generally possess a lower binding affinity towards DNA compared to gold(III) species. A study demonstrated that the binding of the substituted phosphane ligand, 2-(diphenylphosphanoamino)pyridine, to various gold(I) pyridylthiolates, e.g., [Ph_2_PN(H)(2pyr)]AuS(2-pyr) (**42**) (Figure 10), and DNA failed to induce a significant cytotoxic effect and negligible changes were observed in the DNA structure [139] (2-pyr is the anion derived from 2-mercaptopyridine). The evaluation of another phosphanegold(I) thiolate series, Ph_3_PAu[SC(OR)=NC_6_H_4_X-4] for R = Me, Et and iPr, Y = H (**43**) and CH_3_ (**44**) (Figure 10) against HT-29 colorectal cancer cells led to DNA fragmentation, followed by the expression of p53 that led to programmed cell death [56,140]. Over and above these results, gold(I) compounds are also known for their inhibition activity towards enzymes that are involved in DNA replication. Auranofin (**1**), for example, inhibits DNA replication via its interaction with the sulfhydryl groups of DNA polymerases. The presence of a thiolate ligand in auranofin enhances the affinity of this drug towards chalcogenide elements, i.e., sulphide, leading to the formation of an adduct with DNA polymerase that later inhibits the DNA replication process [40,118,133]. In brief, the direct binding of the gold(I) compound towards cancer cell DNA results in the formation of a DNA adduct, which generates a population of damaged DNA cells and further leads to DNA fragmentation when self-repair mechanisms fail to function [141,142,143]. These damaged DNA cells, when arrested at G_2_/M phase, as reported by Tiekink and co-workers, upregulate the pro-apoptotic proteins, p53 or p73, thus initiating the apoptosis pathway to eliminate these DNA damaged cells [56,140].

### 5.2. Inhibition of Topoisomerase

The other major components that play roles in the DNA replication process include DNA topoisomerase I and II, which are actively involved in the modification of the topological state of DNA during replication [144]. The topoisomerase I and II enzymes introduce transient breaks in the phosphodiester backbone of DNA and hence, prevent DNA damage due to supercoiling stress [144]. Owing to the importance of DNA topoisomerases in DNA replication, they are identified as an important target in cancer chemotherapy [145,146]. Tiekink and colleagues showed that phosphanegold(I) thiolates (**43**) (Figure 10) inhibit DNA topoisomerase I at concentrations as low as 2 μM by preventing the enzyme to relax supercoiled pBR322 plasmid DNA [56]. As there is no evidence of the inhibition activity of these gold(I) compounds towards DNA primases, DNA helicases and DNA ligases, it is rather reasonable to propose that DNA functions as the primary target in this case but does not act as the sole contributor for their cytotoxicity. Mitochondria and its protein target, i.e., the thioredoxin reductase enzyme, have emerged as the potential targets for gold compounds, and what is known about their mechanisms will be explored in Section 5.5.

### 5.3. Mitochondria and the Inhibition of Their Function

Mechanistic studies of gold(I) compounds have revealed that mitochondria are the most likely targeted biological sites among several cellular organelles [118]. Mitochondria play an essential role in energy metabolism and in the production of adenosine triphosphate (ATP), oxidative phosphorylation, homeostasis on calcium uptake/release, production of nicotinamide adenine dinucleotide phosphate (NADPH) and the synthesis of DNA [147]. Highly activated mitochondrial activity and the loss of function of mitochondrial inhibitors are the main factors contributing to chemoresistance and poor treatment outcomes for patients undergoing chemotherapy [148]. Therefore, the inhibition of mitochondrial activity through the regulation of the mitochondrial membrane potential and the inhibition of the respective protein activities is essential to determine the induction or inhibition of apoptosis [149]. Instead of targeting the DNA of the host cells, most therapeutic strategies in cancer therapy now target mitochondria as cancer cells present higher mitochondrial activities. Such targeting increases the selectivity of drug candidates and thus, gold-based cytotoxic drugs, with their demonstrated ability to target mitochondria, can also be classified as selective anti-mitochondrial drugs [150].

Based on the anti-cancer activities reported for gold compounds, it can be concluded that the detailed molecular mechanism of gold compounds towards mitochondria is highly dependent on the nature of the ligand, which is closely associated with the propensity to undergo reactions with biological ligands [150]. For instance, binary gold(I) thiolates possess a higher association with cellular components that are rich in sulfhydryl groups [40,151], whereas phosphanegold(I) compounds prefer cellular components with higher contents of thiol-containing proteins or selenium-containing proteins/selenocysteine [118,152,153].

In general, gold(I) thiolates and phosphanegold(I) species function as delocalised lipophilic cations (DLC) and accumulate in the mitochondria of cancerous cells [154,155]. The +I oxidation state of monomeric, neutral and, in general, highly lipophilic gold(I) compounds leads to the direct coordination of “gold” to the selenolate domain of the active site of mitochondrial thioredoxin reductase and further increases the uptake of gold(I) into the mitochondria, which results in swelling and an increased inner mitochondrial membrane potential (IMMP) [15,35]. Following the above-mentioned metabolism, a “point of no return” is passed, resulting in cell death. The membrane of mitochondria comprises two layers, a highly impermeable inner membrane and a permeable outer membrane, with the membrane permeability transition pore (PTP) connecting the inner to the outer mitochondria. The opening of the PTP is strictly regulated by the membrane potential between the membranes. Whereas apoptosis inducing factors (AIFs) are localised within the inner membrane and remain intact under normal conditions, when the cells are exposed to cell death signals, the subsequent increase in ROS activity and increased Bax/Bcl-2 ratio results in the elevation of the outer membrane potential followed by a reduction of the inner membrane potential. A drastic change in the mitochondrial membrane potential results in increased permeability of the outer membrane, allowing the translocation of AIFs from the mitochondria into the cytosol to initiate the apoptosis signaling cascade. More specifically, apoptosis-inducing factors, such as procaspase-9 and cytochrome c, are released from the inter-membrane space to the cytosol, followed by the formation of apoptotic protease activating factor (APAF-1) cell death complexes and the respective cell death signalling cascade, without releasing harmful cell contents to the surrounding tissue [42,156,157]. Unlike the formation of Au–DNA adducts which may not necessary induce cytotoxicity, the gold compounds that affect the mitochondria’s function could potentially cause cytotoxicity towards cancer cells via the mechanism mentioned above [14].

### 5.4. Mitochondrial-Dependent Apoptosis Pathway

The alteration of the mitochondrial membrane potential and the inhibition of thioredoxin reductase induce the accumulation of reactive oxygen species (ROS), such as H_2_O_2_ and oxygen-derived radicals, which promotes lipid peroxidation and the activation of the p38-MAPK apoptosis pathway [158] (MAPK is mitogen-activated protein kinase). Lipid peroxidation, when it occurs, produces lipid radicals, which leads to the dissociation of membrane cytoskeleton and eventually, to cell damage. This process contributes to membrane blebbing which is the hallmark of apoptosis [159]. The activation of the p38-MAPK pathway triggers the activation of initiator caspases such as caspase-3, caspase-7 and caspase-6, as well as the activation of DNase for DNA fragmentation followed by cell death [160].

The other mechanism arising from the depletion of the mitochondrial membrane potential is the regulation of the bax:bcl-2 ratio [161]. When bax is activated and bcl-2 is suppressed, the translocation of bax into the intermembrane space of the mitochondria occurs, which promotes the opening of the membrane pore. As a consequence, the efflux of cytochrome c, procaspase-9 and apoptosis-inducing factors into the cytosol occurs to initiate the mitochondria-dependent pathway of apoptosis [162,163,164].

### 5.5. Inhibition of Thioredoxin

Gold compounds, especially auranofin (**1**), are highly effective at inhibiting the function of the mitochondrial thioredoxin system, which comprises thioredoxin (Trx), thioredoxin reductase (TrxR) and NADPH as the key components [165,166,167]. Under normal conditions, the thioredoxin system participates in cellular processes, such as the reduction of protein disulphide, facilitates the production and removal of ROS, e.g., H_2_O_2_, and regulates the transcription factors required for cell proliferation [168,169,170]. The reduction of Trx by TrxR is an essential event for cancer cells to synthesise transcription factors required for their proliferation, survival, invasion and metastasis [170]. Therefore, the mitochondrial thioredoxin system actually protects cancer cell propagation and contributes to chemoresistance [171,172]. Owing to the above, the mitochondrial thioredoxin system, in general, and thioredoxin reductase, in particular, have become the targets in anti-cancer (and anti-malarial) studies [173,174].

Having the nature of a “soft Lewis acid” and a “thiol-reactive species”, justifies the ability of (**1**) to selectively target the thiol- and selenocysteine-rich thioredoxin system [118,153,175,176]. Species such as (**1**) undergo ligand dissociation to generate various gold(I)-based metabolites and adducts [40,133]. Examples of adducts involved in the cancer cell arrest include (i) thiol–gold(I) adducts that result from the permanent binding of (**1**) to the thiol groups of Trx, and (ii) selenolate–gold(I) adducts produced from the direct binding of (**1**) to the selenocysteine-domain in the active site of TrxR. Adduct formation is accompanied by the loss of thioglucose and leads to irreversible inhibition of TrxR enzymatic activity [139,166,167,177,178,179,180]. The high efficiency of the phosphanegold(I) entity to inhibit Trx and TrxR at nanomolar concentration is mainly due to the high affinity of gold towards chalcogenides, as mentioned above, which promotes the selective and irreversible binding of gold to the selenocysteine domain of TrxR [118,133]. As a consequence of the inhibition of TrxR, the reductase enzyme is converted into an oxidase which results in the high production and accumulation of ROS, followed by lipid peroxidation and oxidative damage to the cells [128,179,181,182]. Due to its ability to directly inhibit Trx activity (i.e., having a different mechanism of action cf. cisplatin), (**1**) is effective at overcoming cisplatin resistance in ovarian cancer cells [179,183]. The inhibition of Trx correlates with the downregulation of the apoptosis signal-regulating kinase (ASK-1) and further regulation of the Bcl-2:Bax ratio (in particular, the inhibition of Bcl-2 gene and activation and further increased activity of the Bax gene) so as to induce an apoptotic cell death pathway via MAP kinase [160,184,185,186].

The structure–activity relationship established for gold(I) compounds that is associated with the inhibition of the thioredoxin reductase enzyme was largely delineated by Gandin and co-workers [77,133,179]. It is noted that while phosphanegold(I) precursors, i.e., Et_3_PAuCl (**45**) and Et_3_PAuBr (**46**), have presented comparable inhibitory effects towards the thioredoxin reductase enzyme at nanomolar concentrations (as exhibited by (**1**)), the coordination of dithiocarbamate and other thiolate ligands has been shown to result in enhanced inhibitory potency. These results are explained in terms of the greater lipophilicity of, for example, phosphanegold(I) dithiocarbamates, enabling them to readily permeate the cell membrane to interact with the thioredoxin or selenocysteine domain of the active site of thioredoxin reductase [133]. Ott et al. reported that (**45**), when subjected to cellular uptake against the HT-29 colorectal carcinoma cell line, showed only 10% of transportation into the nucleus for reaction with DNA, with the remaining 90% hypothesised to react with non-DNA targets, such as mitochondria [5].

**Figure 11:**
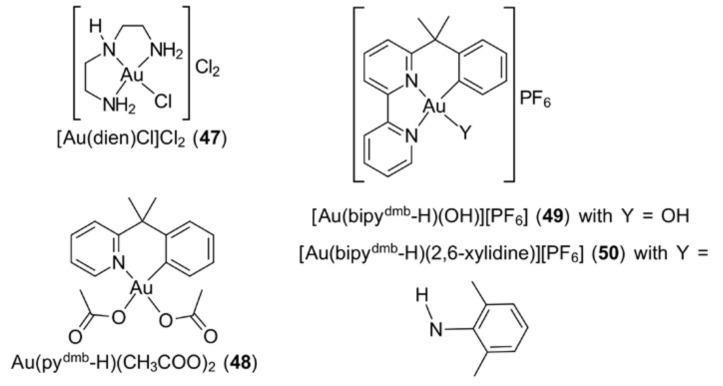
Chemical structures of (**47**)–(**50**).

In contrast, gold(III) congeners have been less explored in this context. Despite being reported, in 1998, that gold(III) complexes require a 1000-fold higher concentration than gold(I) drugs to yield inhibition effects on TrxR [187], several gold(III) complexes (i.e., [Au(dien)Cl]Cl_2_ (**47**), Au(py^dmb^-H)(CH_3_COO)_2_ (**48**), [Au(bpy^dmb^-H)(OH)][PF_6_] (**49**) and [Au(bpy^dmb^-H)(2,6-xylidine)][PF_6_] (**50**); Figure 11) have displayed promising inhibitory dosages in the micromolar range, while Au(S_2_CNMe_2_)X_2_ (**40**) and Au[S_2_CN(Me)CH_2_C(=O)OEt]X_2_ (**41**), X = Cl and Br, have shown inhibition of TrxR at nanomolar concentrations [132,188,189]. The inhibitory potency of gold compounds is largely influenced by the oxidation state of their metal centres and the lability of coordinated ligand(s). Gold(III), being a ‘hard’ metal (cf. gold(I)), presents a relatively lower probability for coordination with the selenocysteine active site of TrxR [24,118,190].

The inhibition of thioredoxin (Trx) results in the suppression of cancer cell invasion and metastasis [191]. When cell proliferation is suppressed, the inhibition of NF-κB genes in cancer cells occurs, followed by the downregulation of the vascular endothelial growth factor (VEGF) and matrix metalloproteinase-9 (MMP-9); MMP-9 aids in the degradation of the extracellular matrix of blood vessels and thus, reduces the rate of cancer cell invasion and migration [191,192,193].

## 6. Conclusions

The stability of gold-based drugs under physiological condition remains a challenge for the development of effective therapeutic agents. The nature of the ligand coordinated to the gold centre greatly determines the pharmacokinetic profiles of both gold(I) and gold(III) compounds. Compared with platinum-based complexes, gold compounds with oxidation states of +I and +III possess better selectivity and potency towards cancer cells than normal cells due to their weaker DNA-binding activity and greater affinity towards the sulfhydryl, thiol and selenocysteine groups of several protein targets. In short, the biological benefits of gold compounds are prompted from a series of complex interactions between metal, cellular components and genes. However, like many other drugs, gold compounds induce deleterious toxic side effects and future research, based on experimental trials, augmented by in silico studies, e.g., molecular docking studies, needs to be directed towards selective targeting of cancer cells to enhance the effectiveness of these compounds and to minimise unwanted biological responses. In summary, based on the exciting results revealed thus far, more extensive investigation is clearly justified to fully assess the potential anti-cancer and toxicity profiles of gold-based compounds.
